# Supplementary material for: Treatment of VLCAD-Deficient Patient Fibroblasts with Peroxisome Proliferator-Activated Receptor δ Agonist Improves Cellular Bioenergetics
Source: Cells. 2022 Aug 24;11(17):2635. doi: 10.3390/cells11172635 (PMC9454759; doi:10.3390/cells11172635)
Supplement: Supplementary file 1 [file cells-11-02635-s001.zip › cells-1831137-supplementary.pdf]

Supplementary Information

Treatment of VLCAD deficient patient fibroblasts with peroxisome-proliferator activated receptor δ agonist improves cellular bioenergetics

Olivia M. D'Annibale[1,2], Yu Leng Phua[1], Clinton Van't Land[1], Anuradha Karunanidhi[1], Reneo junior co-author[3], Al-Walid Mohsen[1,2], Reneo senior co-author[3], Jerry Vockley[1,2]

[1] Division of Genetic and Genomic Medicine, Department of Pediatrics, University of Pittsburgh School of Medicine, and UPMC Children's Hospital of Pittsburgh, Pittsburgh, PA 15224;

[2] Department of Human Genetics, University of Pittsburgh Graduate School of Public Health, Pittsburgh, PA 15261;

[3]Reneo Pharmaceuticals, XXX, CA, USA

Supplemental Information, Tables

Supplementary Table S1. Oligonucleotide sequence of primers used in qPCR experiments and corresponding Primer Bank ID.

Gene	Primer Bank ID	Forward	Reverse	Forward TM	Reverse TM	Product Size
ACADVL	764964473c1	ACAGATCAGGTGTTCCCATACC	CTTGGCGGGATCGTTCACTT	61.2	62.5	114
HADHA	105990523c2	CTGCCCAAAATGGTGGGTGT	GGAGGTTTTAGTCCTGGTCCC	63	61.5	134
HADHB	105990524c1	CTGTCCAGACCAAAACGAAGAA	CGATGCAACAAACCCGTAAGC	60.4	62.4	160
ETFDH	119703745c1	TACTGTGCCTCGAATTACTACCC	ACAGCCAACTGTTTTAGACGAA	61.2	60.1	165
UQCRC2	50592987c1	TTCAGCAATTTAGGAACCACCC	GGTCACACTTAATTTGCCACCAA	60.5	61.5	120
NDUFS2	260898742c2	GCTGTTATGTACCCAAGCAAAGA	TCCCCACTCAATTCCATCACT	60.8	60.5	164
TOMM20	208609996c1	GGTACTGCATCTACTTCGACCG	TGGTCTACGCCCTTCTCATATTC	62.1	61.2	220

Supplementary Table S1. Oligonucleotide sequence of primers used in qPCR experiments and corresponding Primer Bank ID.

Forward and reverse primer sets listed are in Table S1 were obtained from Primer Bank.

Supplementary Information, Figures

Supplementary Figure S1. ChIPseq analysis for PPARδ utilizing HUVEC cells.

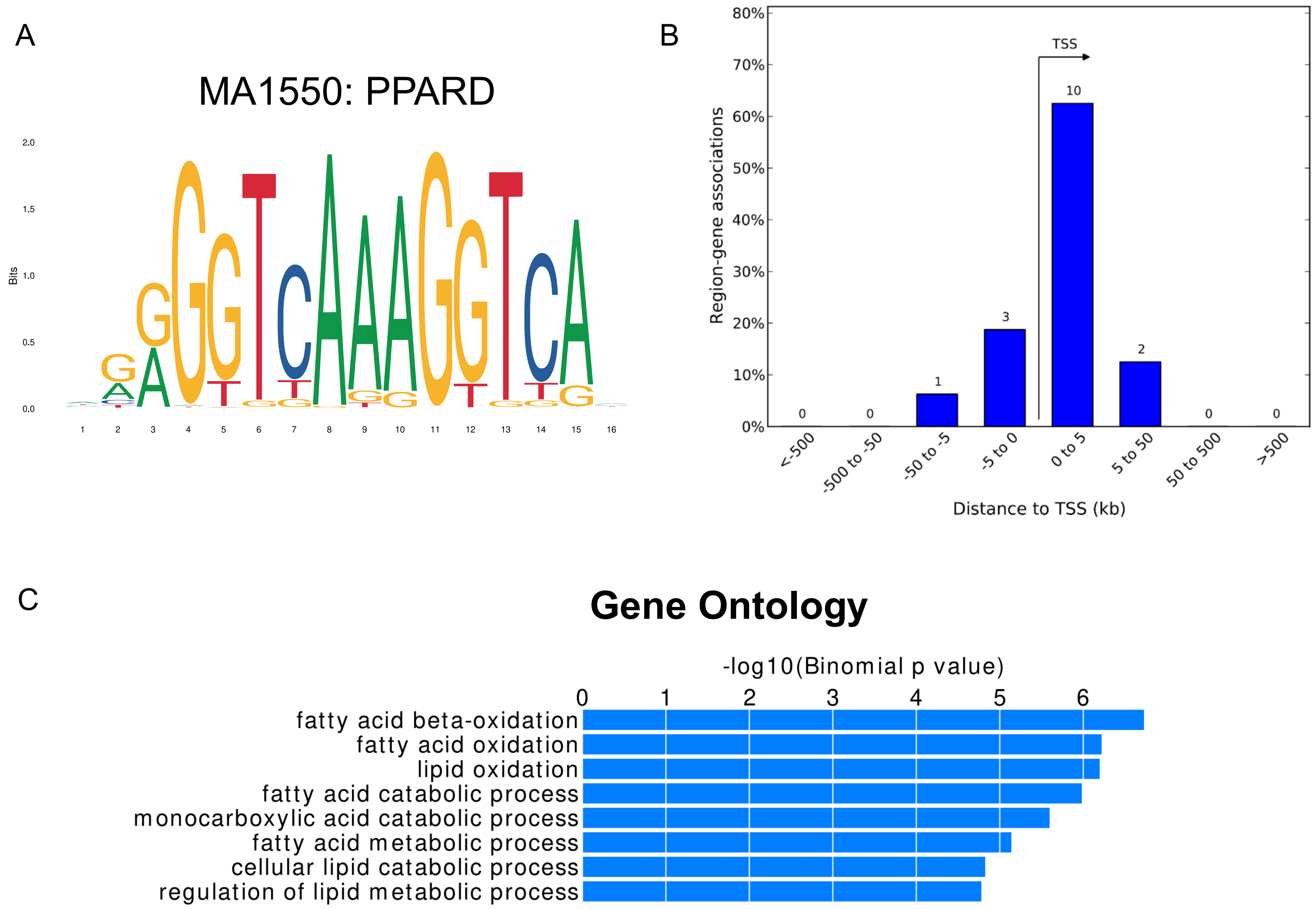

D

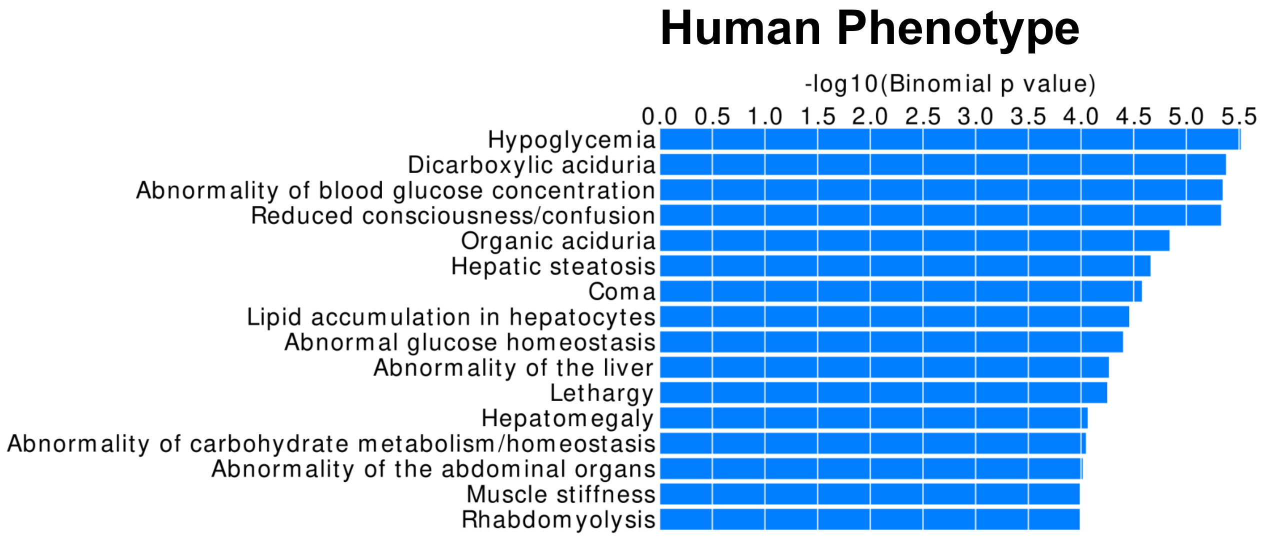
Human Phenotype
-log10(Binomial p value)
0.0 0.5 1.0 1.5 2.0 2.5 3.0 3.5 4.0 4.5 5.0 5.5
Hypoglycemia
Dicarboxylic aciduria
Abnormality of blood glucose concentration
Reduced consciousness/confusion
Organic aciduria
Hepatic steatosis
Coma
Lipid accumulation in hepatocytes
Abnormal glucose homeostasis
Abnormality of the liver
Lethargy
Hepatomegaly
Abnormality of carbohydrate metabolism/homeostasis
Abnormality of the abdominal organs
Muscle stiffness
Rhabdomyolysis

E

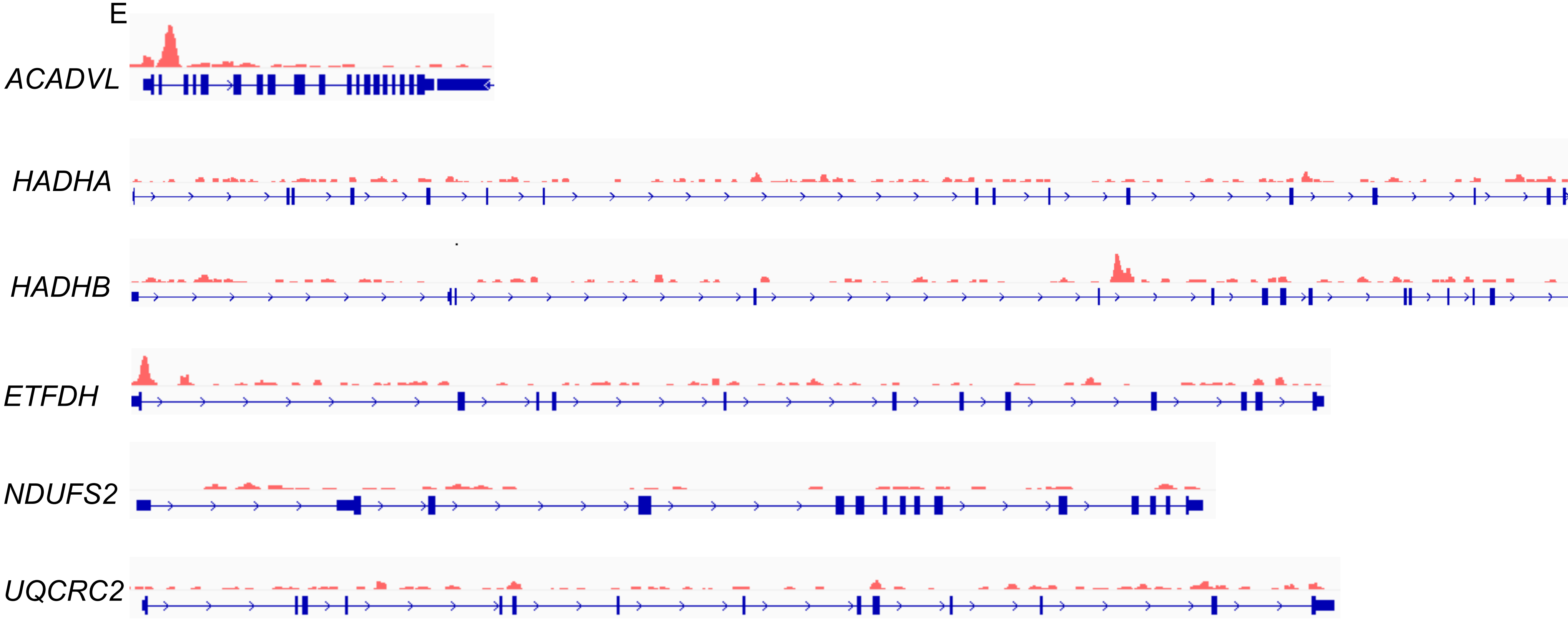
ACADVL
HADHA
HADHB
ETFDH
NDUFS2
UQCRC2

Supplementary Figure S1. ChIPseq analysis for PPARdelta utilizing HUVEC cells. MACS2 analysis of PPARδ ChIPseq identified a localized intergenic region with a consensus binding motif. Larger letters indicate more highly conserved residues (A). PPARD is a primary DNA enhancer identified 5kb downstream of the transcription start site (TSS) (B). The fatty acid metabolism pathway is enriched by PPARδ target genes (C) and target genes (D) are implicated in FAO disorder phenotypes at high levels of statistical significance as indicated by the binomial p-value on the x-axis. Enrichment of PPARδ binding sites in the genome are indicated by the ChIP-seq data as indicated by the prominent peaks (red) located ~ 5 kb downstream of transcription start site of the coding genes. The coding exons and non-coding regions are indicated by the vertical and horizontal blue bars respectively. Of note, both *ACADVL* and *ETFDH* demonstrated a higher enrichment of PPARδ binding at the transcription start site.

(E).

Supplementary Figure S2. Western blot of control and VLCADD fibroblasts treated with REN001.

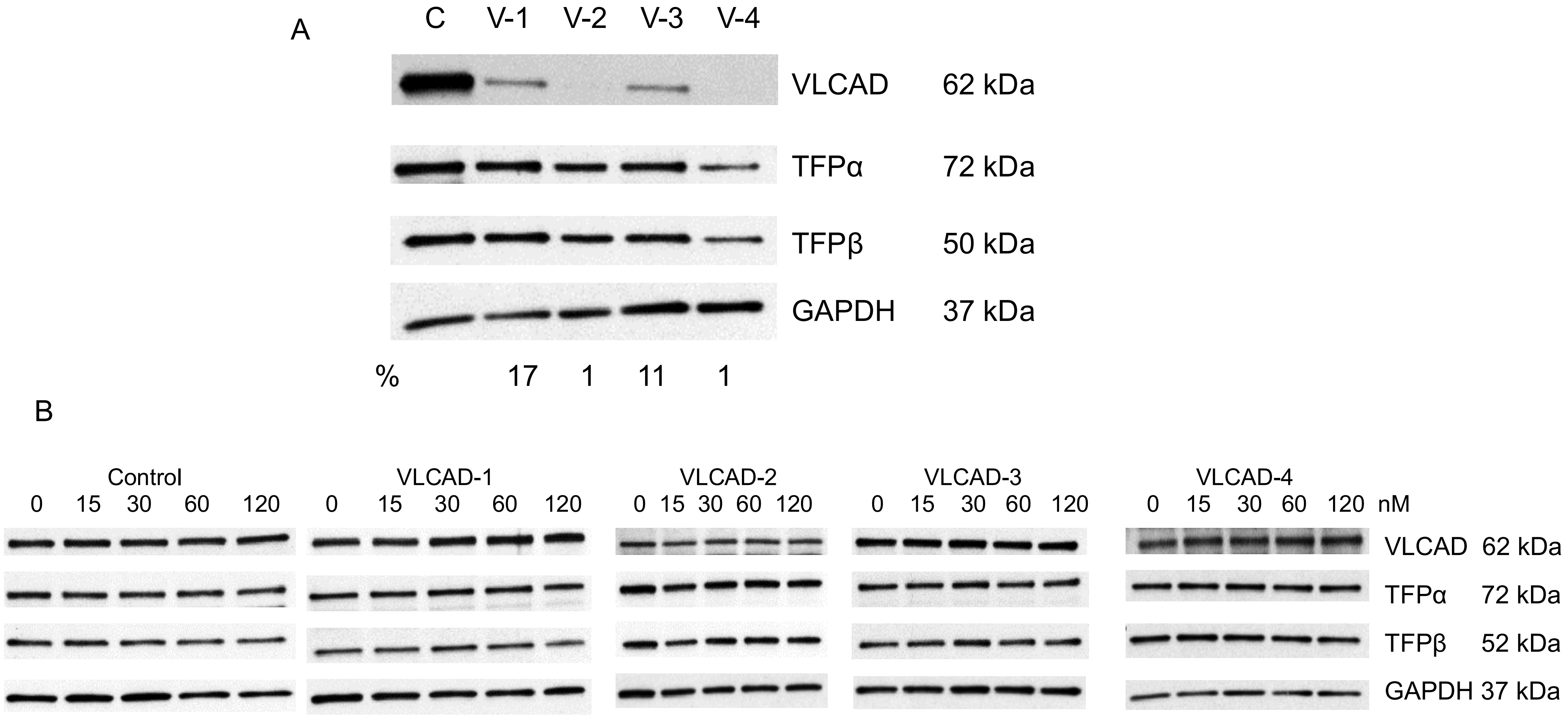

Supplementary Figure S2. Western blot of control and VLCADD fibroblasts treated with REN001. Control and four VLCADD fibroblasts blotted for VLCAD, TFPα, TFPβ, and GAPDH (A). Fibroblasts were treated with 0, 15, 30, 60, and 120 nM of REN001 for 48 hours and harvested (B). 25 μg of protein was loaded into each well and membranes were probed for VLCAD, TFPα, TFPβ, and

GAPDH as a loading control. Overexposures were taken of VLCADD cell lines to better capture the bands and to quantify. Blots were repeated 3 times. Band intensity was quantified using ImageLab (BioRad).

Supplementary Figure S3. Immunofluorescence and quantification of control fibroblasts treated with REN001.

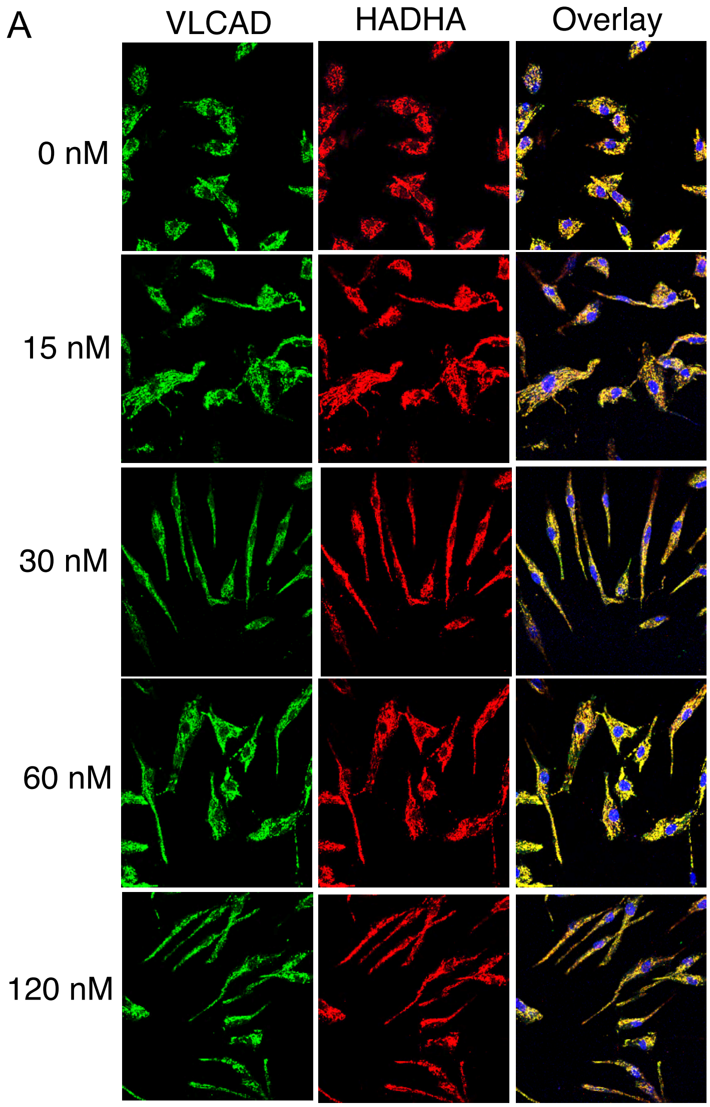

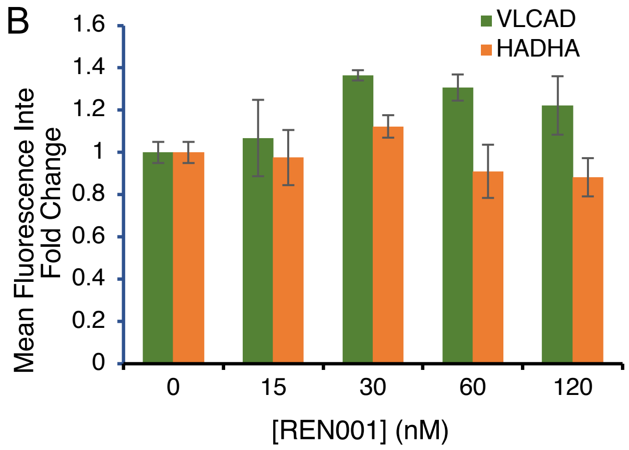

Supplementary Figure S3. Immunofluorescence and quantification of control fibroblasts treated with REN001.

Control fibroblasts were treated with REN001 in complete DMEM for 48 hours. Treated fibroblasts were fixed and stained with VLCAD (green) and HADHA (red) antibodies. Nuclei were stained with Dapi. Quantification was performed via ImageJ (B). Data are presented as fold changes compared to itself at 0 nM treatment (n = 6 for all assays).

Supplementary Figure S4. Evaluation of VLCAD and MCAD enzyme activity in VLCADD whole cell lysates treated with REN001.

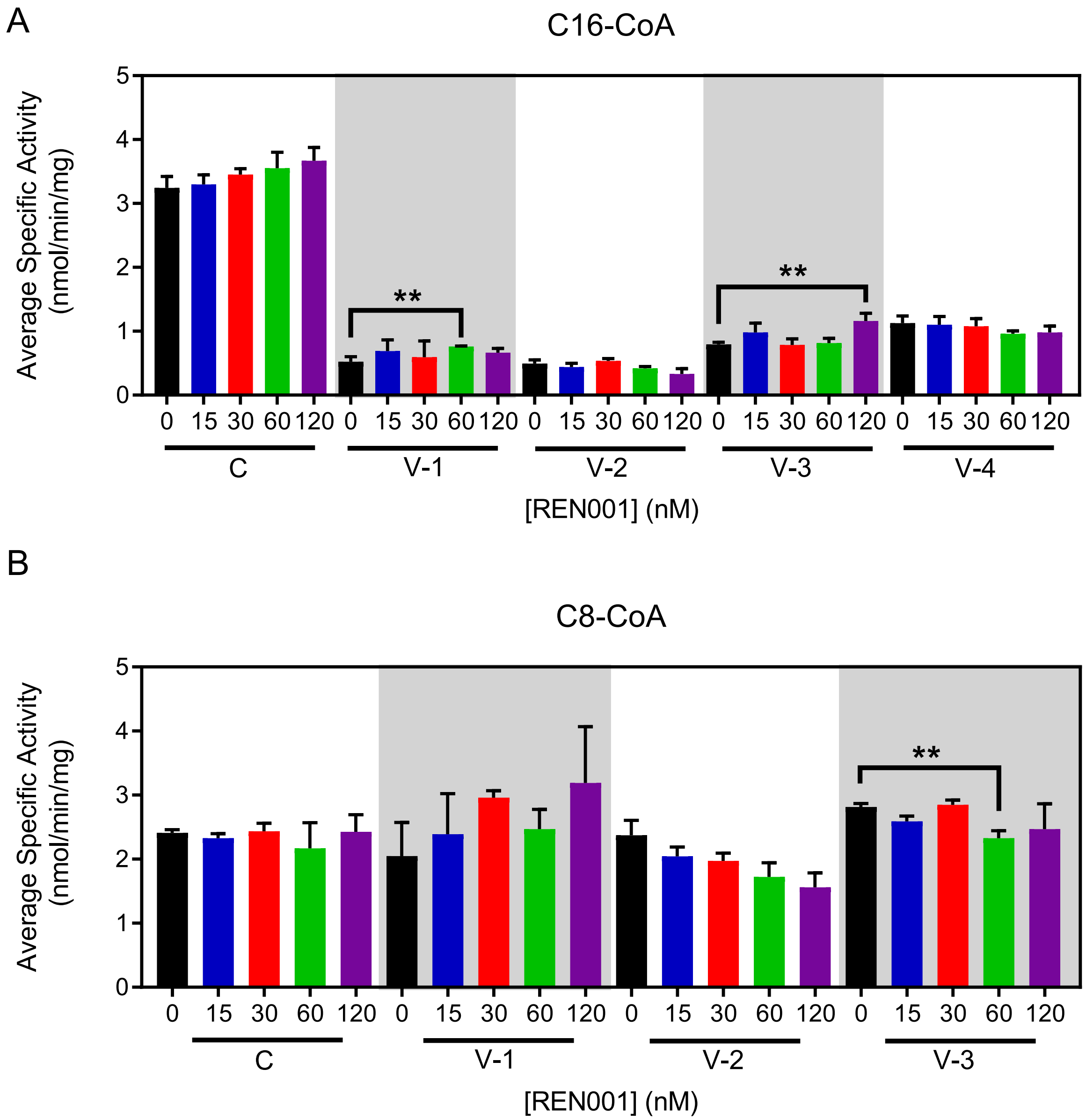

Supplemental Figure S4. Evaluation of VLCAD and MCAD enzyme activity in VLCADD whole cell lysates treated with REN001. Activity was measured using C16-CoA as substrate (A). Bars represent means and standard deviations in triplicate assays. Activity was measured using

C8-CoA as the substrate (B). V-4 (VLCAD-4) was not measured for MCAD activity due to minimal sample. Bars represent mean and standard deviations in triplicate assays. *$p < 0.05$, **$p < 0.01$, compared to itself at

0 nM treatment (*t* test for unpaired samples).

Supplemental Figure S5. Oxygen consumption rate of control and VLCAD deficient cell lines treated with REN001.

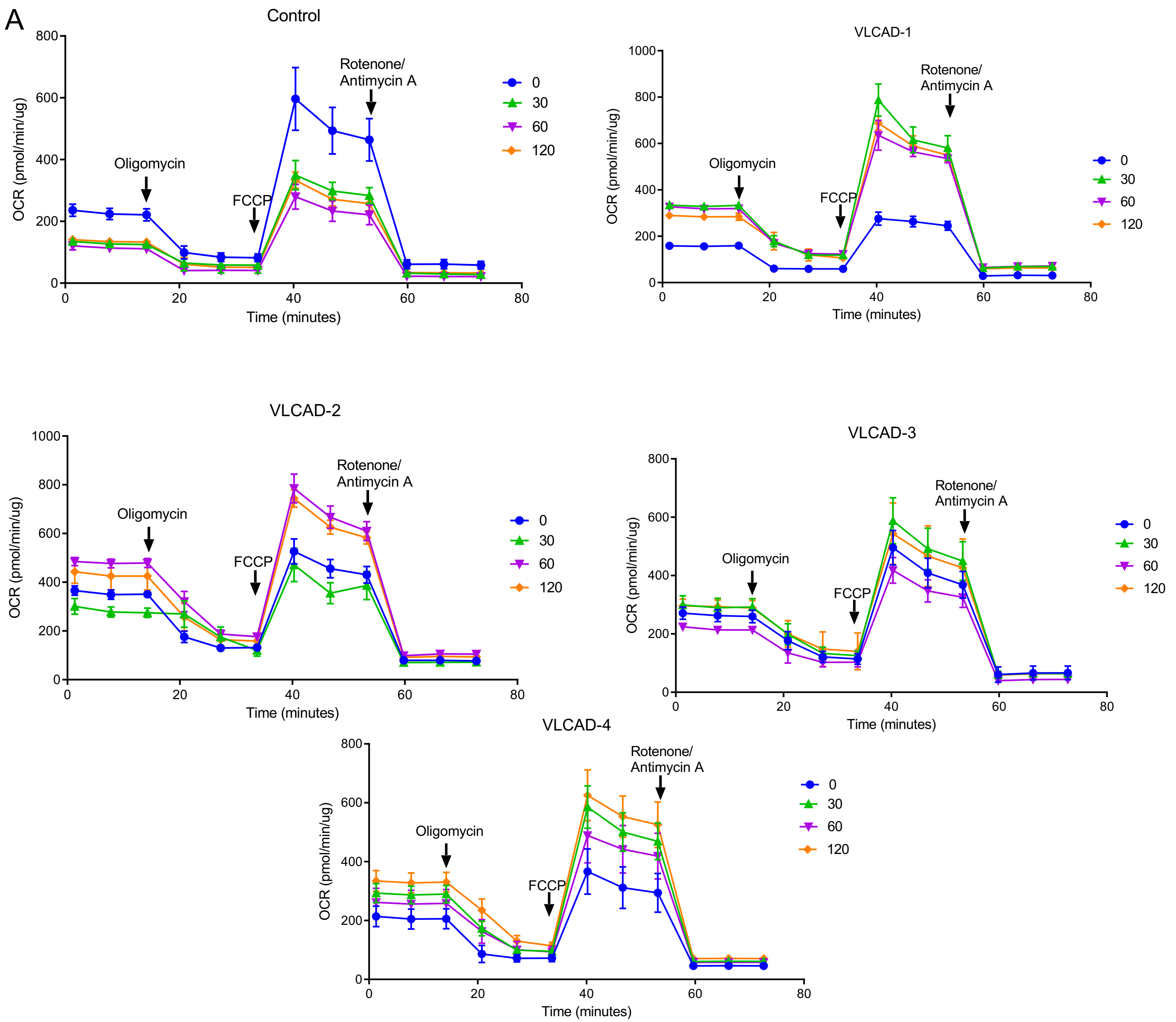

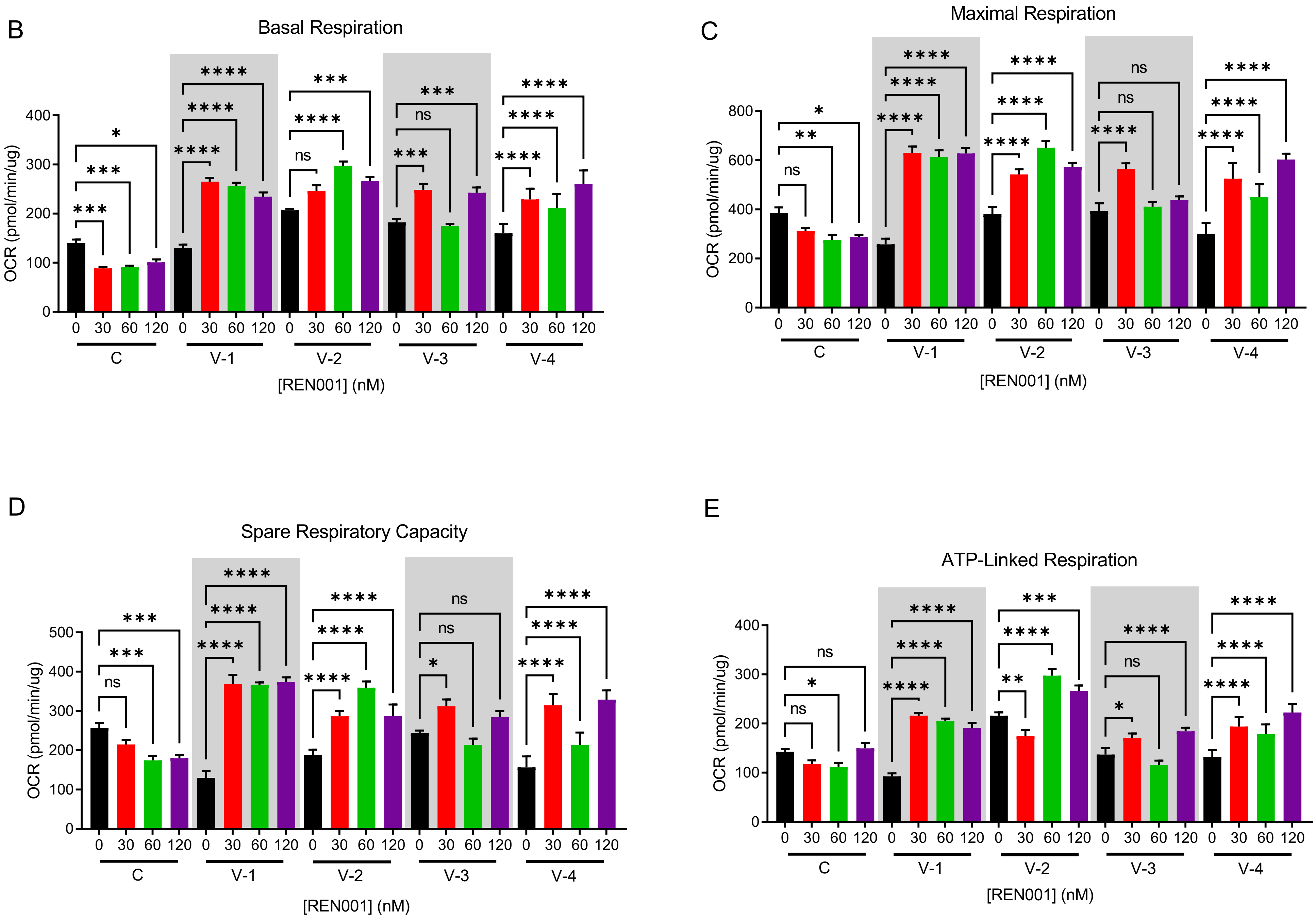
B
Basal Respiration
OCR (pmol/min/ug)
0
100
200
300
400
ns
*
***
****
0 30 60 120
C
V-1
V-2
V-3
V-4
[REN001] (nM)
C
Maximal Respiration
OCR (pmol/min/ug)
0
200
400
600
800
ns
*
**
****
[REN001] (nM)
D
Spare Respiratory Capacity
OCR (pmol/min/ug)
0
100
200
300
400
500
ns
*
***
****
[REN001] (nM)
E
ATP-Linked Respiration
OCR (pmol/min/ug)
0
100
200
300
400
ns
*
**
***
****
[REN001] (nM)

Supplementary Figure S5. Oxygen consumption rate of control and VLCAD deficient cell lines treated with REN001. Oxygen consumption rate (OCR) was measured in the resting state (basal respiration) followed by injection of oligomycin (ATP synthase inhibitor) that reduces OCR, representing ATP turnover. Subsequent inject of FCCP dissipates the proton gradient and allows maximum respiration. Finally, rotenone and antimycin A are added to completely disable the electron transport chain, inhibiting the total mitochondrial respiration. The remaining OCR represents non-mitochondrial respiration (A). Control and VLCADD cell lines were treated with REN001 for 48 hr. Basal respiration (B), maximal respiration (C), spare respiratory capacity (D), and ATP production (E). Bars represent mean and standard deviations of 6-8 wells per parameter. *$p < 0.05$, **$p < 0.01$, ***$p <0.001$, ****$p < 0.0001$, compared to itself at 0 nM treatment ($n = 6$ for all samples; *t* test for unpaired samples).

Supplemental Figure S6. Oxygen consumption rate of control and VLCAD deficient cell lines treated with Bezafibrate.

A

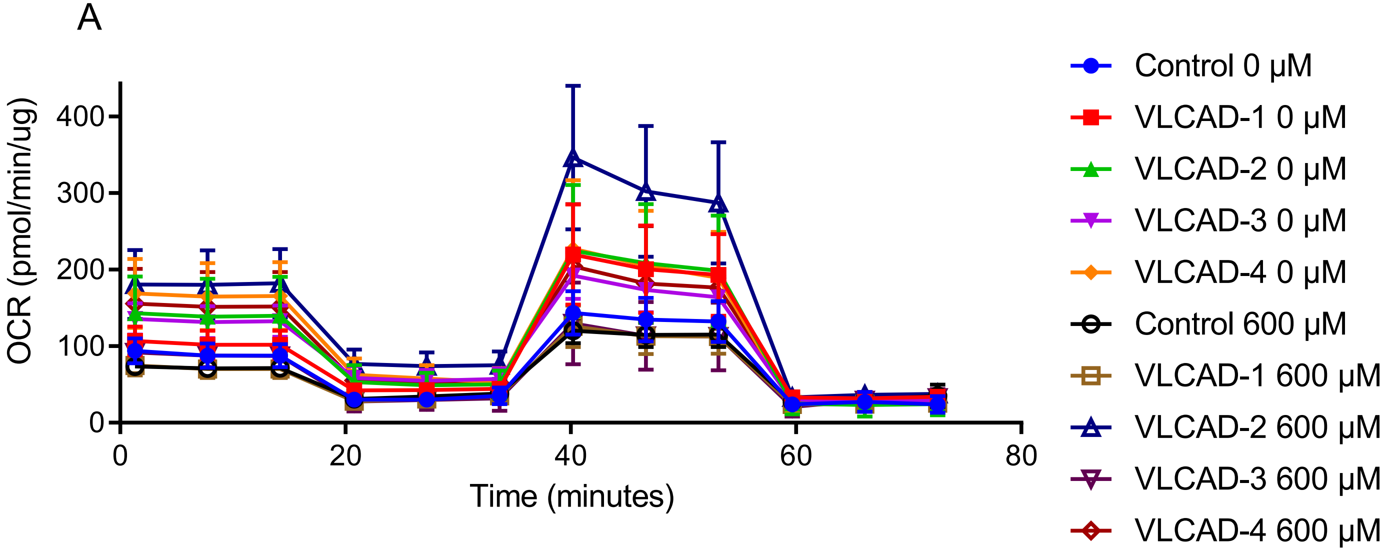

Supplemental Figure S6. Oxygen consumption rate of control and VLCAD deficient cell lines treated with Bezafibrate. Oxygen consumption rate (OCR) was measured in the resting state (basal respiration) followed by injection of oligomycin (ATP synthase inhibitor) that reduces OCR, representing ATP turnover. Subsequent inject of FCCP dissipates the proton gradient and allows maximum respiration. Finally, rotenone and antimycin A are added to completely disable the electron transport chain, inhibiting the total mitochondrial respiration. The remaining OCR represents non-mitochondrial respiration (A). Control and VLCADD cell lines were treated with bezafibrate for 48 hr.

Supplemental Figure S7. Acylcarnitine profiling of control and VLCADD fibroblasts treated with REN001.

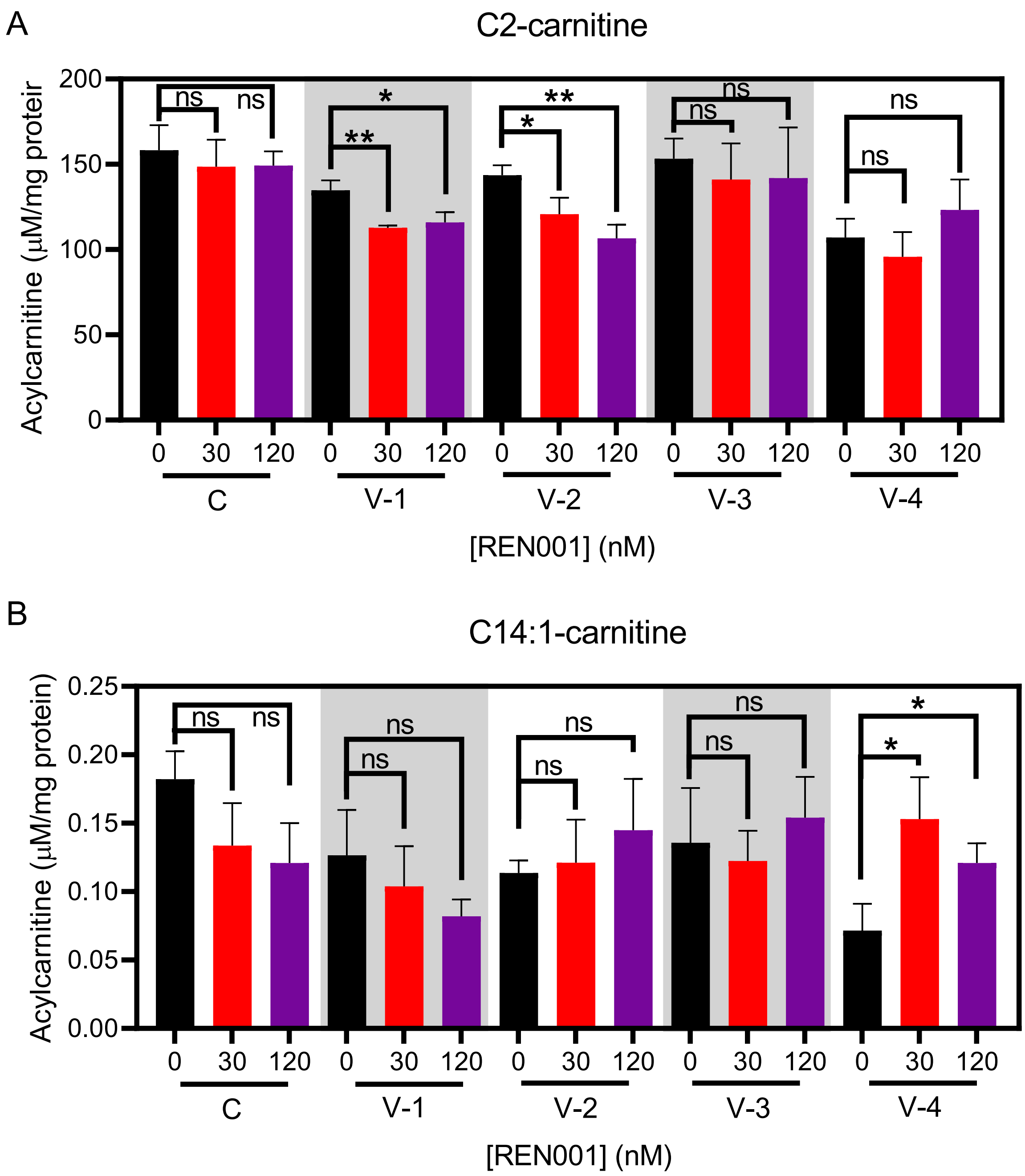

C

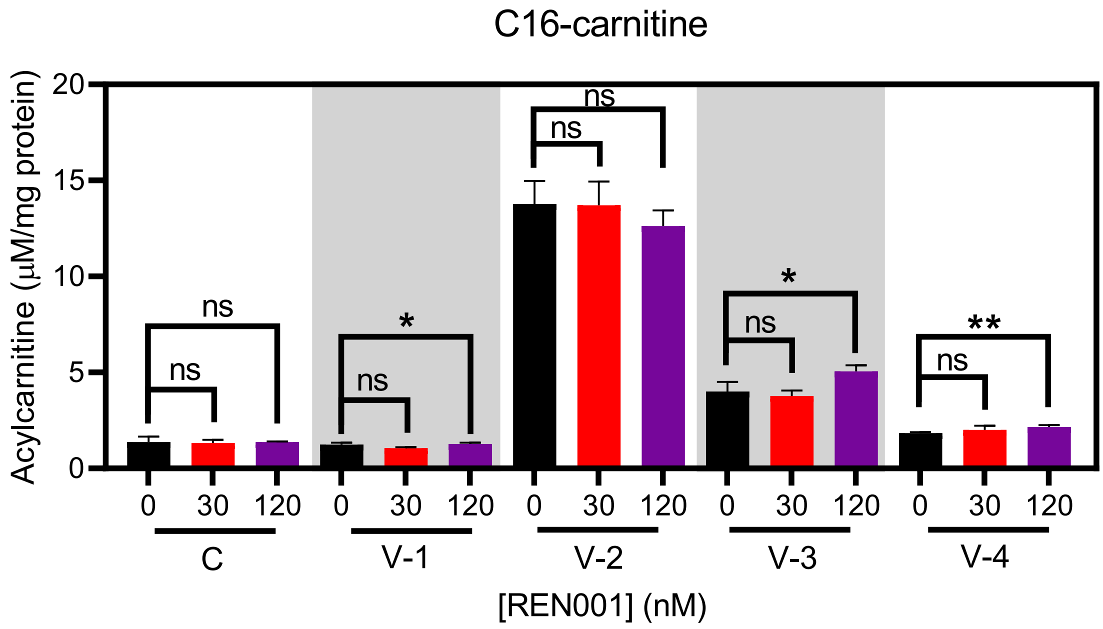

Supplemental Figure S7. Acylcarnitine profiling of control and VLCADD fibroblasts treated with REN001. Media was collected after 72 hr incubation and analyzed via tandem mass spectrometry. Acetylcarnitine (C2-carnitine) (A), C14:0-carnitine (B), and palmitoylcarnitine (C16-carnitine) (C). Bars represent mean and standard deviations in triplicate assays. *p < 0.05, **p < 0.01, ns = not statistically significant, compared to itself at 0 nM treatment (n = 3 samples for all assays; *t* test for unpaired samples).
